# Author Correction: Acute stress induces long-term metabolic, functional, and structural remodeling of the heart

**DOI:** 10.1038/s41467-023-39910-7

**Published:** 2023-07-12

**Authors:** Thulaciga Yoganathan, Mailyn Perez-Liva, Daniel Balvay, Morgane Le Gall, Alice Lallemand, Anais Certain, Gwennhael Autret, Yasmine Mokrani, François Guillonneau, Johanna Bruce, Vincent Nguyen, Umit Gencer, Alain Schmitt, Franck Lager, Thomas Guilbert, Patrick Bruneval, Jose Vilar, Nawal Maissa, Elie Mousseaux, Thomas Viel, Gilles Renault, Nadjia Kachenoura, Bertrand Tavitian

**Affiliations:** 1 Université Paris Cité, Inserm, PARCC, F-75015 Paris, France; 2 Nuclear Physics Group and IPARCOS, Department of Structure of Matter, Thermal Physics and Electronics, CEI Moncloa, Universidad Complutense de Madrid, 28040 Madrid, Spain; 3 Université Paris Cité, Plateforme d’Imageries du Vivant, PARCC, F-75015 Paris, France; 4 Université Paris Cité, P53 proteom’IC facility, Institut Cochin, INSERM, CNRS, F-75014 Paris, France; 5 Institut de Cancérologie de l’Ouest, CNRS UMR6075 INSERM U1307, 15 rue André Boquel, F-49055 Angers, France; 6 Sorbonne Université, Laboratoire d’Imagerie Biomédicale, Inserm, CNRS, F-75006 Paris, France; 7 Service de Radiologie, AP-HP, hôpital européen Georges Pompidou, F-75015 Paris, France; 8 Université Paris Cité, Cochin Imaging, Electron microscopy, Institut Cochin, INSERM, CNRS, F-75014 Paris, France; 9 Université Paris Cité, Plateforme d’Imageries du Vivant, Institut Cochin, Inserm-CNRS, F-75014 Paris, France; 10 Université Paris Cité, Cochin Imaging Photonic, IMAG’IC, Institut Cochin, Inserm, CNRS, F-75014 Paris, France

**Keywords:** Cardiovascular biology, Imaging, Cardiovascular diseases

Correction to: *Nature Communications* 10.1038/s41467-023-39590-3, published online 28 June 2023

In this article the affiliation ‘Nuclear Physics Group and IPARCOS, Department of Structure of Matter, Thermal Physics and Electronics, CEI Moncloa, Universidad Complutense de Madrid, 28040 Madrid, Spain’ for Mailyn Perez-Liva was missing.

In addition, the funding from ‘the Programme Ramón y Cajal RYC2021-032739-I, funded by MCIN/AEI/10.13039/501100011033 and the European Union “NextGenerationEU”/PRTR’ for Mailyn Perez-Liva was omitted.

The original article has been corrected.

