# Supplementary material for: Acute stress induces long-term metabolic, functional, and structural remodeling of the heart
Source: Nat Commun. 2023 Jun 28;14:3835. doi: 10.1038/s41467-023-39590-3 (PMC10307856; doi:10.1038/s41467-023-39590-3)
Supplement: Supplementary file 1 — Supplementary Information [file 41467_2023_39590_MOESM1_ESM.pdf]

Acute stress induces long-term metabolic, functional, and structural remodeling of the heart

SUPPLEMENTARY INFORMATION

Supplementary Methods

2D Echocardiography

High resolution echocardiography was performed in B-mode and M-mode using a high-frequency ultrasound transducer (18 MHz, VEVO2100; Visualsonics). During the imaging sessions, rats were kept under isoflurane anesthesia with constant monitoring of heart and respiration rate and body temperature. Pulsed Doppler mode was used to measure transmitral blood flow velocities.

In vivo left coronary artery flow reserve measurements

Coronary artery flow reserve (CFR) was measured with transthoracic US Doppler, using a high concentration of isoflurane (5% rate mixed in 100% air) as vasodilator **(CFR, extended data 2)**. The pulsed Doppler probe was positioned over the proximal left anterior descending coronary artery and the coronary flow spectrum was characterized as a biphasic flow. The peak diastolic velocity at baseline (2% of isoflurane) and during hyperemia (5% of isoflurane over 5min) was measured, and the CFR was calculated as the ratio of the hyperemic to basal peak diastolic velocity.

Electrocardiogram (ECG)

ECG signals were recorded using 2 positive electrodes placed on the surface of the fore paws and 1 negative electrode placed on the surface of its left hind paw (PowerLab, ADInstruments, Sydney, Australia).

Noninvasive blood pressure measurement

Blood pressure was measured in the rat tail using the volume pressure recording sensor technology (tail-cuff CODA® High Throughput system, Kent Scientific).

Immunohistochemistry (IHC)

Each cardiac section was stained in triplicate for Periodic Acid Shiff (PAS) and oil-red. PAS and oil-red stainings were performed manually. Stained sections were scanned in their entirety with a NanoZoomer HT 2.0 (Hamamatsu) at a magnification of x20.

Second Harmonic Generation (SHG)

SHG and TPEF (Two-Photon Excitation Fluorescence) images were acquired with a nonlinear multiphoton Leica SP8 DIVE microscope (Leica microsystems Gmbh, Wetzlar, Germany) coupled with a Coherent Discovery (Coherent Inc., Santa Clara, CA, USA) laser source. Three fields of view were acquired from paraffin embedded 4 µm-thick sections of apical and basal LV regions. To quantify SHG signal, the microscope settings were adjusted using a non-fibrotic control heart section. TPEF and SHG images were acquired with a 25x W 0.95 NA objective, excitation wavelength was 1040 nm at constant power and photomultiplier tubes and hybrid detectors were used at 800 V and 80% gain respectively. SHG score was proceeded using a homemade ImageJ macro from the IMAG'IC core facility.

Transmission electron microscopy

Apical and basal segments of the LV were fixed in Trump's fixative solution (#50 980 239, ThermoFisher), a combination of sodium cacodylate, formalin and glutaraldehyde, and dehydrated. Samples (1 cubic mm) were immersed twice in propylene oxide baths, in a prepolymer/propylene oxide mix, then left in a pure prepolymer, and finally embedded in gelatin capsules. Capsules were polymerized during at least 24 hours in an oven at 60°C. Samples were sectioned with a Reichert Ultracut S ultramicrotome. Semi-fine sections were taken to check the quality and orientation of the sample. They were then stained with toluidine blue and stored. Ultra-fine sections (90 nm thick) were cut with a diamond knife (Diatome). The ultrastructure images were observed with a transmission electron microscope JEOL 1011, (JEOL ltd, Tokyo, Japan) using the camera GATAN Orius (AMETEK,

USA). Images were acquired with software Digital Micrograph® (AMETEK, USA) and exported in uncompressed tiff format.

BNP plasmatic dosage

BNP plasmatic concentrations were measured by ELISA (RayBio Human/Mouse/Rat BNP enzyme Immunoassay kit, Norcross, Georgia, USA) according to the manufacturer's protocol. All samples were assayed in duplicate.

Supplementary figures

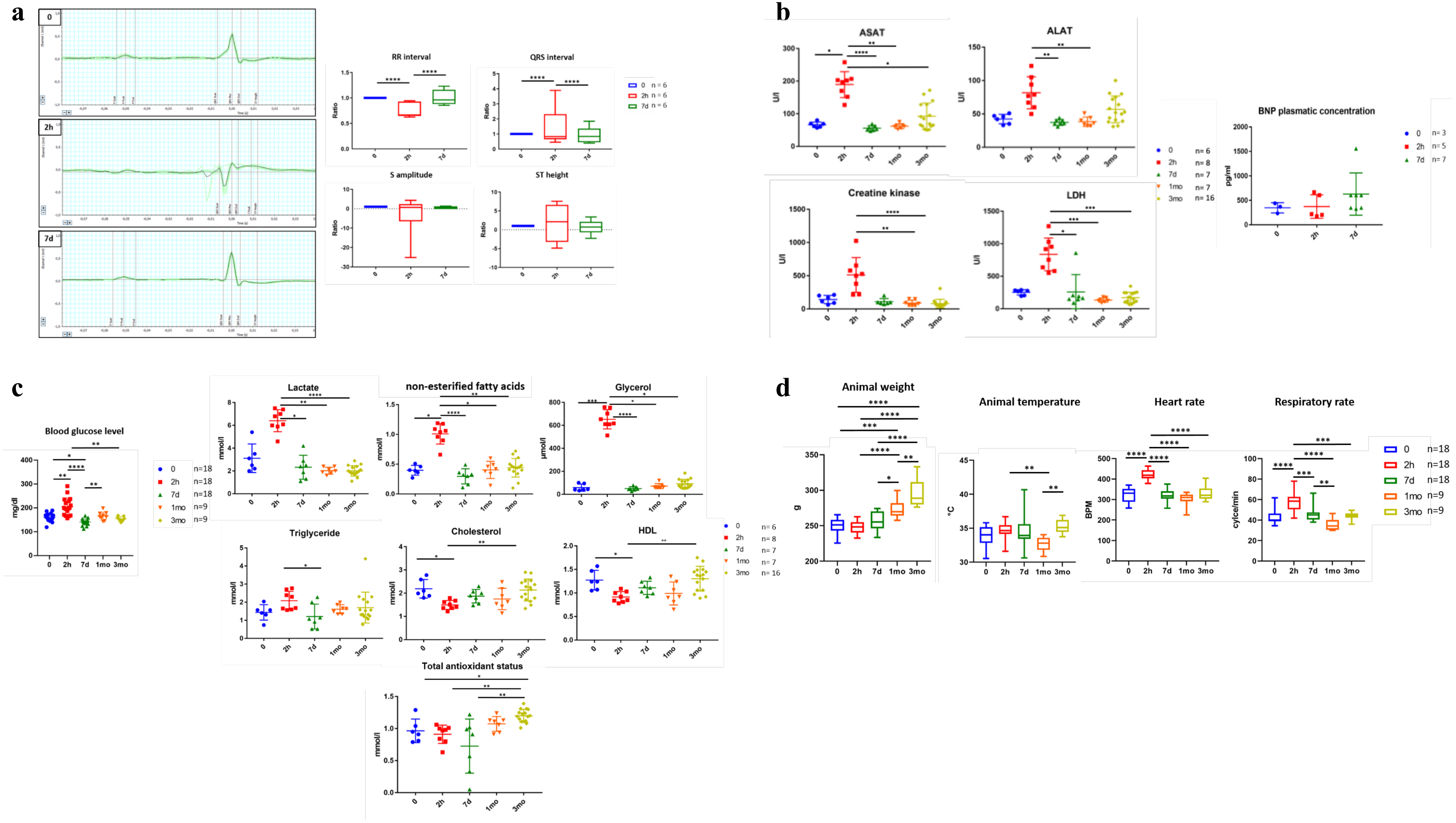
a
0
2h
7d
RR interval
QRS interval
S amplitude
ST height
Ratio
0 n = 6
2h n = 6
7d n = 6
b
ASAT
ALAT
Creatine kinase
LDH
BNP plasmatic concentration
U/l
pg/ml
0 n= 6
2h n= 8
7d n= 7
1mo n= 7
3mo n= 16
0 n= 3
2h n= 5
7d n= 7
c
Blood glucose level
mg/dl
0 n=18
2h n=18
7d n=18
1mo n=9
3mo n=9
Lactate
non-esterified fatty acids
Glycerol
Triglyceride
Cholesterol
HDL
Total antioxidant status
mmol/l
µmol/l
0 n= 6
2h n= 8
7d n= 7
1mo n= 7
3mo n= 16
d
Animal weight
Animal temperature
Heart rate
Respiratory rate
g
°C
BPM
cylce/min
0 n=18
2h n=18
7d n=18
1mo n=9
3mo n=9

Supplementary figure 1. Effect of isoprenaline on electrocardiogram, plasmatic constants, and physiological constants. a Typical signs of acute stress were observed on the boxplots showing median, 25 and 75 percentiles, and extremes of electrocardiograms values of rats recorded 2h post intraperitoneal injection of isoprenaline (ISO, 50 mg/kg), notably a shortening of the RR interval (0 vs. 2h: p<0.0001 and 2h vs. 7d: p<0.0001) and a longer QRS interval (0 vs. 2h: p<0.0001 and 2h vs. 7d: p<0.0001). No statistically significant changes were found in the amplitude of the S wave or the height of the ST segment, although these parameters were highly variable during the acute-stress phase. **b** Plasmatic biomarkers of heart failure ASAT (0 vs. 2h: p=0.0377, 2h vs. 7d: p<0.0001, 2h vs. 1mo: p=0.0029 and 2h vs. 3mo: p=0.0478), ALAT (2h vs. 7d: p=0.0019, and 2h vs. 1mo: p=0.0034), Creatine kinase (2h vs. 1mo: p=0.0038, and 2h vs. 3mo: p<0.0001) and LDH (2h vs. 7d: p=0.0228, 2h vs. 1mo: p=0.0003, and 2h vs. 3mo: p=0.0003) increased during the catecholaminergic surge and later returned to normal, while a modest increase of BNP was measured at 7d post stress. **c** The plasmatic concentrations of energy substrates glucose (0 vs. 2h: p=0.0076, 0 vs. 7d: p=0.0274, 2h vs. 7d: p<0.0001, 2h vs. 3mo: p=0.0044, and 7d vs. 1mo: p= 0.0274), lactate (2h vs. 7d: p=0.0160, 2h vs. 1mo: p=0.0022, and 2h vs. 3mo: p<0.0001), non-esterified fatty acids (0 vs. 2h: p=0.0151, 2h vs. 7d: p<0.0001, 2h vs. 1mo: p=0.0173, 2h vs. 3mo: p= 0.0097), glycerol (0 vs. 2h: p=0.0004, 2h vs. 7d: p<0.0001, 2h vs. 1mo: p=0.0164, and 2h vs. 3mo: p=0.0290), and triglyceride (2h vs. 7d: p= 0.0209) augmented at 2h post-ISO and then returned to normal. In contrast, cholesterol (0 vs. 2h: p=0.0200, and 2h vs. 3mo: p=0.0080) and HDL (0 vs. 2h: p=0.0482, and 2h vs. 3mo: p=0.0043) were lower at 2h and returned to pre-ISO values afterwards, while the total antioxidant status (0 vs. 3mo: p= 0.0455, 2h vs. 3mo: p=0.0026, and 7d vs. 3mo: p=0.0031) was unchanged except at the latest time point. **d** Boxplots showing median, 25 and 75 percentiles, and extremes of basic physiological parameters values: ISO increases heart rate (0 vs. 2h: p<0.0001, 2h vs. 7d: p<0.0001, 2h vs. 1mo: p<0.0001, and 2h vs. 3mo: p<0.0001) and respiratory rate (0 vs. 2h: p<0.0001, 2h vs. 7d: p=0.0008, 2h vs. 1mo: p<0.0001, 2h vs. 3mo: p=0.0006, and 7d vs. 1mo: p=0.0035) during the acute phase only, body weight gain (0 vs. 1mo: p=0.0011, 0 vs. 3mo: p=0.0002, 2h vs. 1mo: p=0.0002, 2h vs. 3mo: p= <0.0001, 7d vs. 1mo: p=0.0044, 7d vs. 3mo: p=<0.0001, and 1mo vs. 3mo: p=0.0002) and body temperature (2h vs. 1mo: p=0.0104, and 1mo vs. 3mo: p=0.0011) are unaffected by ISO. Unpaired comparison tests: * p<0.05, ** p<0.01 and *** p<0.001. Statistical significance (p<0.05) for each variable was estimated by one-way or two-way ANOVA when group variances were equal (Bartlett test); if not the non-parametric Kruskall-Wallis test, and the Holm multiple comparisons test was used to execute simultaneous t-tests. Source data are provided as a Source Data file.

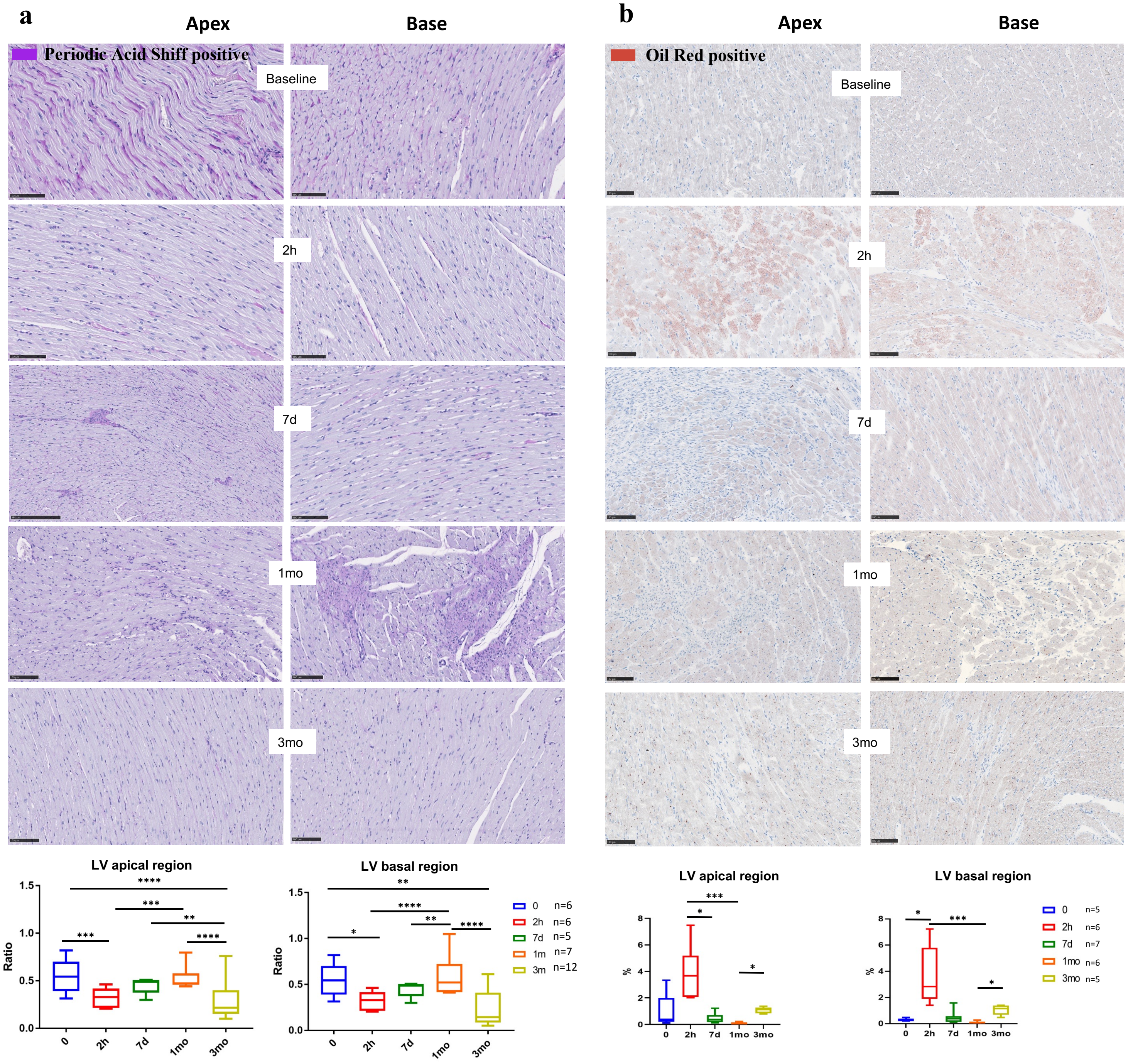
a
Apex
Base
Periodic Acid Shiff positive
Baseline
2h
7d
1mo
3mo
LV apical region
LV basal region
Ratio
0
2h
7d
1mo
3mo
0 n=6
2h n=6
7d n=5
1m n=7
3m n=12
b
Apex
Base
Oil Red positive
Baseline
2h
7d
1mo
3mo
LV apical region
LV basal region
%
0 n=5
2h n=6
7d n=7
1mo n=6
3mo n=5

Supplementary figure 2. Cardiac reserves of energy substrates post-ISO. a Representative sections stained for glycogen by Periodic acid Schiff (PAS) at the indicated time points post-ISO represented in boxplots showing median, 25 and 75 percentiles, and extremes of values. Sections for the apex and base obtained from the same animal at each time point. Box plots for the indicated number of animals show a loss of glycogen deposits in the myocardium at 2h and at 3mo post-ISO both in the apex (0 vs. 2h: p=0.0005, 0 vs. 3mo: p<0.0001, 2h vs. 1mo: p=0.0002, 7d vs. 3mo: p=0.0325, and 1mo vs. 3mo: p<0.0001) and the base of the heart (0 vs. 2h: p=0.0186, 0 vs. 3mo: p=0.0048, 2h vs. 1mo: p<0.0001, 7d vs. 1mo: p=0.0011, and 1mo vs. 3mo: p<0.0001). **b** Representative sections stained for lipid droplets by Oil Red at the indicated time points post-ISO represented in boxplots showing median, 25 and 75 percentiles, and extremes of values. Sections for the apex and base obtained from the same animal at each time point. Box plots for the indicated number of animals show a transient increase of lipid droplets in the myocardium at 2h post-ISO both in the apex (0 vs. 2h: p=0.1994, 2h vs. 7d: p=0.0379, 2h vs. 1mo: p=0.0001, and 1mo vs. 3mo: p=0.0436) and the base (0 vs. 2h: p=0.0482, 2h vs. 1mo: p<0.0001, and 1mo vs. 3mo: p=0.0385) of the heart. Unpaired comparison tests: * p<0.05, ** p<0.01 and *** p<0.001. Statistical significance (p<0.05) for each variable was estimated by one-way or two-way ANOVA when group variances were equal (Bartlett test); if not the non-parametric Kruskall-Wallis test, and the Holm multiple comparisons test was used to execute simultaneous t-tests. Scale bars in the images correspond to a length of 100 μm. Source data are provided as a Source Data file.

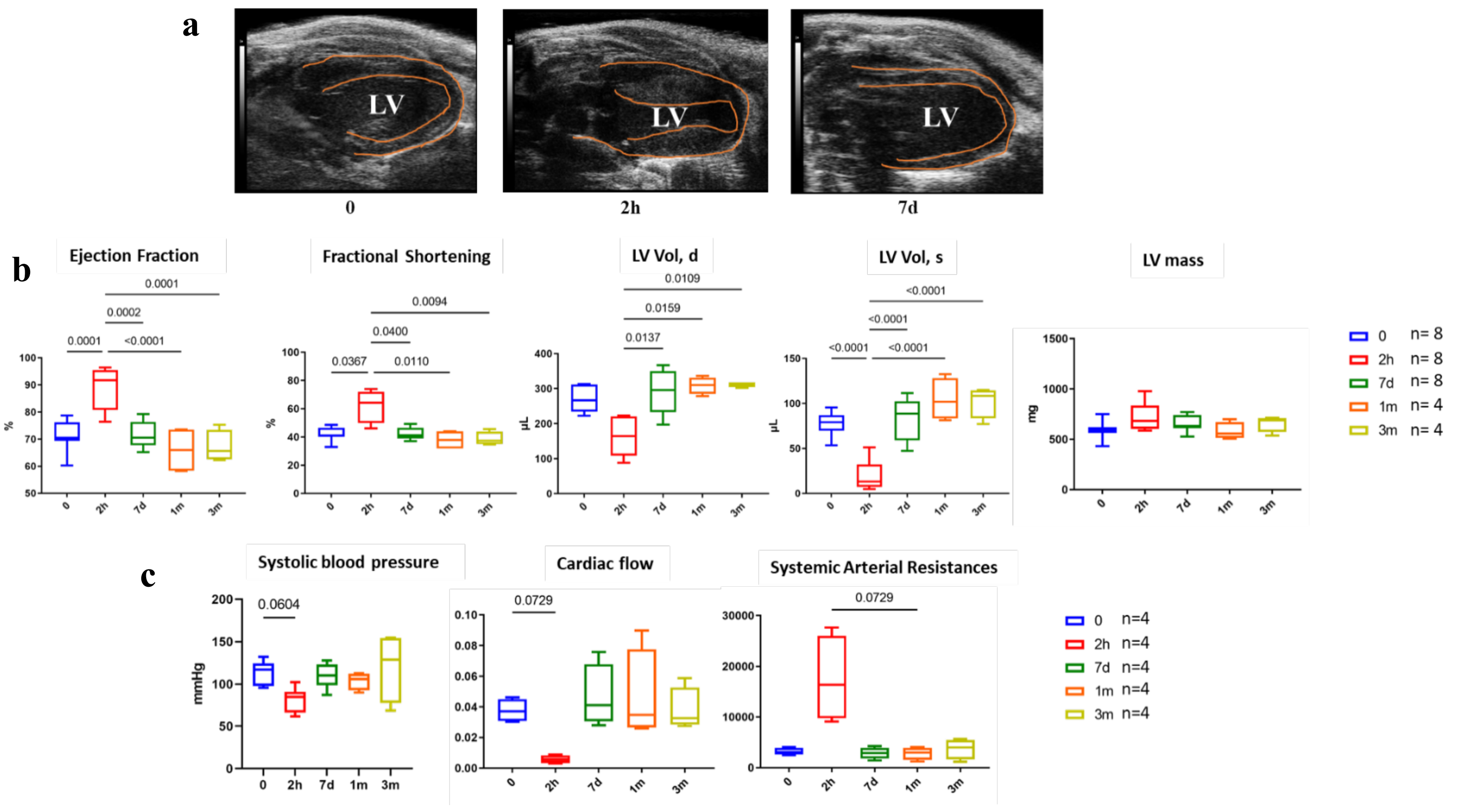

Supplementary figure 3. High resolution echocardiography. a Representative echocardiographic images of the LV of one animal showing a drastic reduction of the diastolic volume at 2h post ISO, followed by recovery at 7d post-ISO. **b** Boxplots showing median, 25 and 75 percentiles, and extremes of echocardiographic parameters at the indicated time points post-ISO. At 2h, the increase in the ejection fraction and fractional shortening are typical of the reduced LV volume during the diastolic (d) and systolic (s) phases. Both return later to normal. LV mass remains unchanged. **c** During the acute stress phase, systolic blood pressure and cardiac flow were reduced while systemic arterial resistances were increased, expected by the effect of ISO. Values represented in boxplots showing median, 25 and 75 percentiles, and extremes of values did not reach significance due to the small number of animals (n=4). All parameters recovered pre-ISO values by 7d post-ISO. Unpaired comparison tests. Statistical significance ($p<0.05$) for each variable was estimated by one-way or two-way ANOVA when group variances were equal (Bartlett test); if not the non-parametric Kruskall-Wallis test, and the Holm multiple comparisons test was used to execute simultaneous t-tests. Source data are provided as a Source Data file.

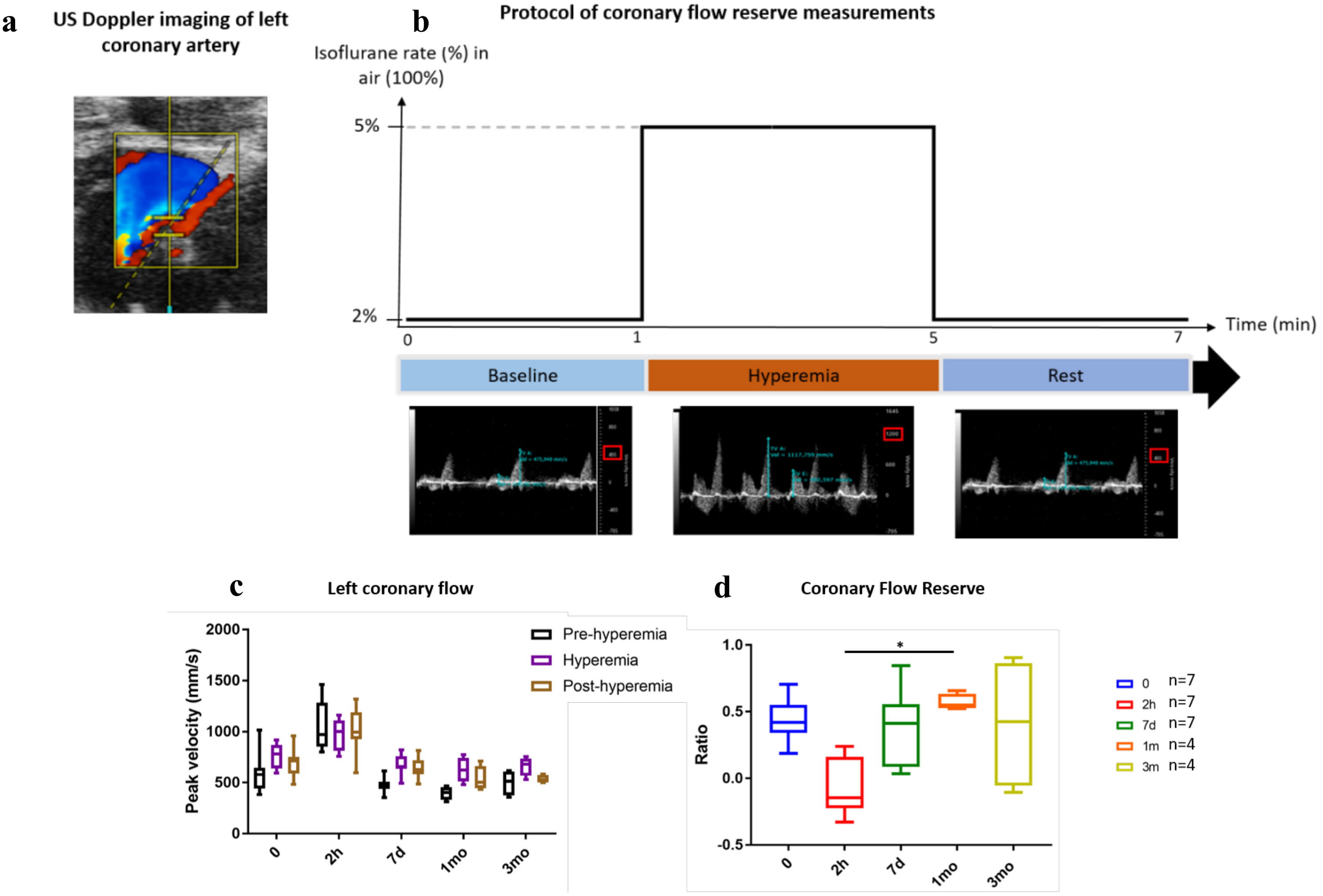

Supplementary figure 4. Left coronary flow reserve measurements experiments. a Ultrasound Doppler imaging of a left coronary artery. **b** Protocol describing the induction of hyperemia using isoflurane. **c** LV coronary flow (CF, peak velocity in mm per second) in pre-hyperemic, hyperemic, and post-hyperemic conditions at baseline (n=8 animals) and 2h (n=8 animals), 7d (n=8 animals) and 1- (n=4 animals) and 3-months (n=4 animals) post-ISO, represented in boxplots showing median, 25 and 75 percentiles, and extremes of values. During the catecholaminergic surge, pre-hyperemic CF increases 1.7-fold. However, the CF is already maximal and not further increased by hyperemia. After the catecholaminergic surge, pre-hyperemic, hyperemic, and post-hyperemic CF return to pre-ISO values. **d** Time course of the coronary flow reserve (CFR = (CF during hyperemia – CF pre-hyperemia)/ CF pre-hyperemia) represented in boxplots showing median, 25 and 75 percentiles, and extremes of values depicting reduction at 2h post-ISO followed by recovery (2h vs. 1mo: p=0.0495). Unpaired comparison tests: * p<0.05, ** p<0.01 and *** p<0.001. Statistical significance (p<0.05) for each variable was estimated by one-way or two-way ANOVA when group variances were equal (Bartlett test); if not the non-parametric Kruskall-Wallis test, and the Holm multiple comparisons test was used to execute simultaneous t-tests. Source data are provided as a Source Data file.

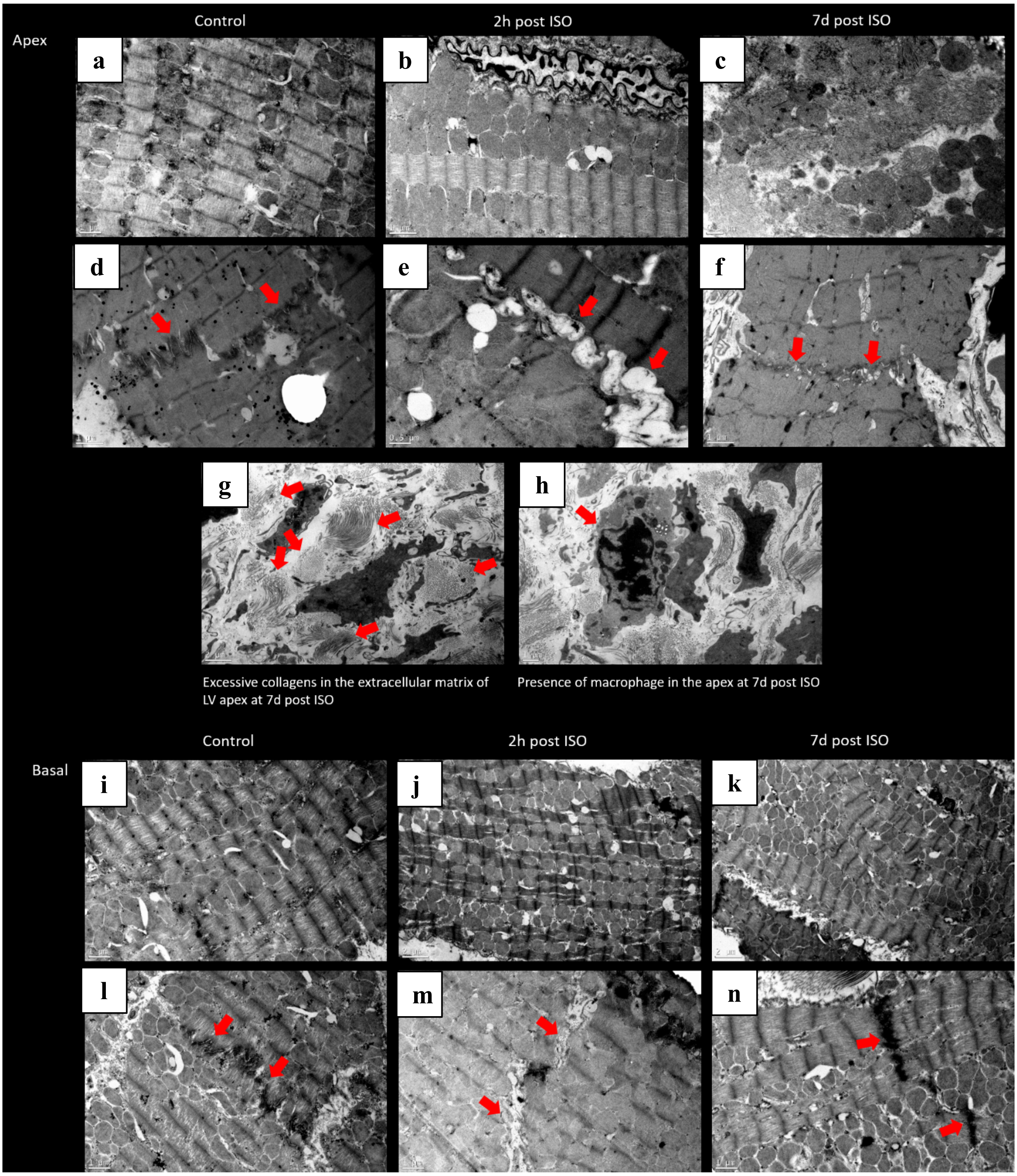
Control
2h post ISO
7d post ISO
Apex
a
b
c
d
e
f
g
h
Excessive collagens in the extracellular matrix of LV apex at 7d post ISO
Presence of macrophage in the apex at 7d post ISO
Control
2h post ISO
7d post ISO
Basal
i
j
k
l
m
n

Supplementary figure 5. Transmission electron microscopy (TEM) of the heart ultrastructure at baseline, 2h, and 7 days post-ISO. Representative micrographs from n=5 hearts, in control (no-ISO), 2h and 7d post-ISO: **a-h** apical section, **i-k**: basal section of the heart. **a-c** Organization of the sarcomere fibers in the heart apex: normal orientation and alignment in the control group and at 2h post-ISO. **c** The normal alignment of cardiac myofibers was no longer recognizable in some fields of view at 7d post-ISO, while this was never observed pre-ISO or at 2h post-ISO. **d-f** The intercalated discs in the apex had a normal appearance in the control group but appeared disrupted at 2h post-ISO, suggesting a mechanical stress induced by ISO-induced hypercontractility. At 7d post-ISO intercalated discs were normal. **g** Important presence of collagens in the extracellular matrix at 7d post-ISO. **h** Every now and then, a phagocytic macrophage was observed at 7d post-ISO. No collagen deposits or macrophages were observed in the no-ISO control or the 2h post-ISO group. i-k Normal alignment and orientation of fibers in the basal region in the control group and at 2h and 7d post-ISO. **l-n** As in the apex, basal intercalated discs appeared transiently disrupted at 2h post-ISO and recovered their normal appearance afterwards.

Scale bars in images **a, b, d, f, h, i, l, m** and **n** correspond to a length of 1 μm. Scale bars in images **c** and **e** correspond to a length of 0.5 μm. Scale bars in images **g, j** and **k** correspond to a length of 2 μm.

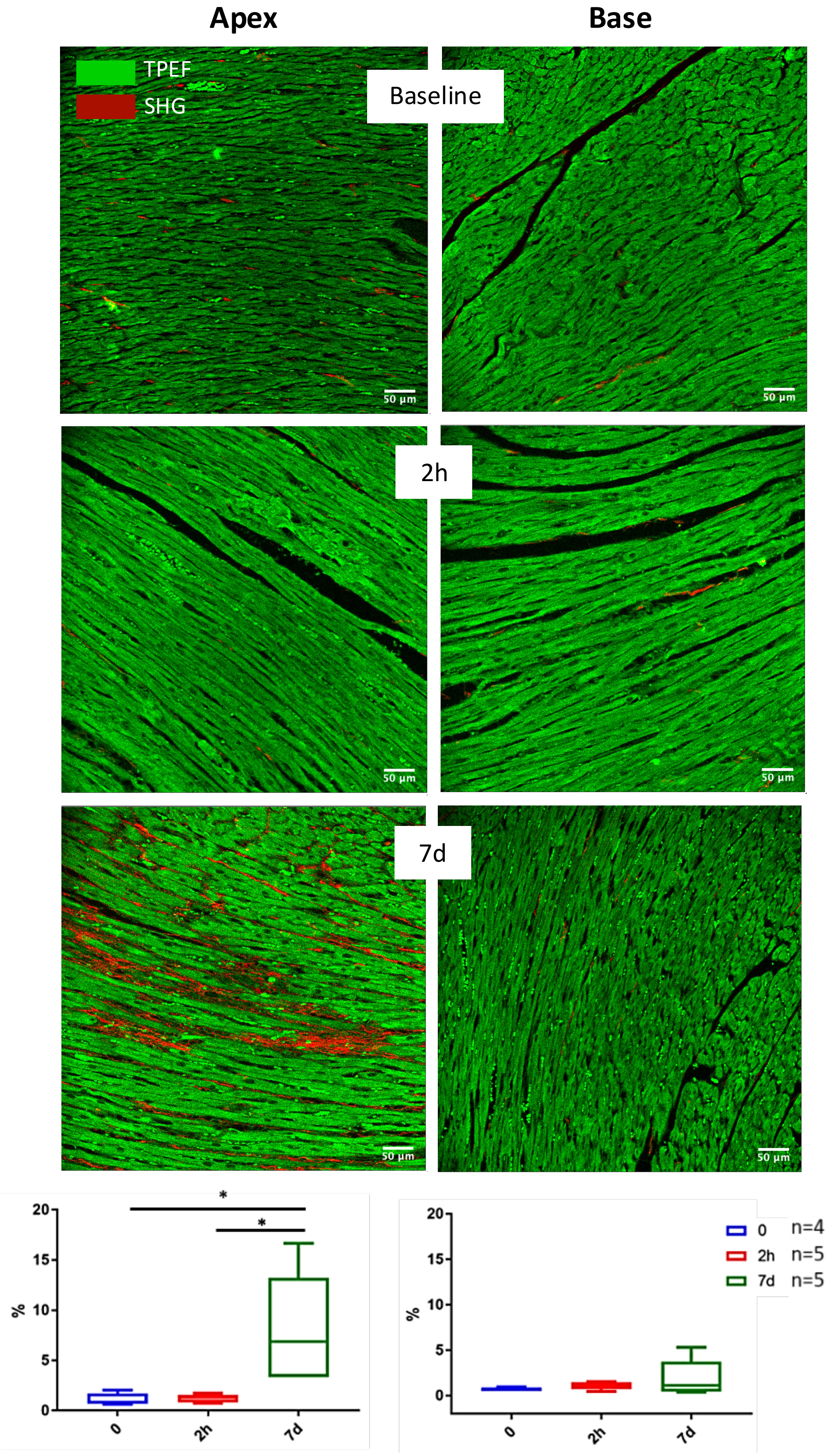
Apex
Base
TPEF
SHG
Baseline
2h
7d
50 µm
%
0
2h
7d
*
*
n=4
n=5
n=5

Supplementary figure 6. Second Harmonic Microscopy of cardiac tissue and SHG scores. Representative fluorescence micrographs of n=3 fields-of-view per section from 8 sections (5 in the apex and 3 in the base) of n= 4 (baseline) and n=5 (2h and 7d post-ISO) hearts. TPEF: two-photon excited auto-fluorescence of the tissue, SHG: collagen-specific signal. Boxplots showing median, 25 and 75 percentiles, and extremes of values of the SHG score depicted a clear augmentation at 7d post-ISO in the apex (0 vs. 7d: $p=0.0173$, and 2h vs. 7d: $p=0.0467$), indicating a significant increase in cardiac collagen synthesis. Statistical significance ($p<0.05$) for each variable was estimated by one-way or two-way ANOVA when group variances were equal (Bartlett test); if not the non-parametric Kruskall-Wallis test, and the Holm multiple comparisons test was used to execute simultaneous t-tests. Scale bars in the images correspond to a length of 50 μm.

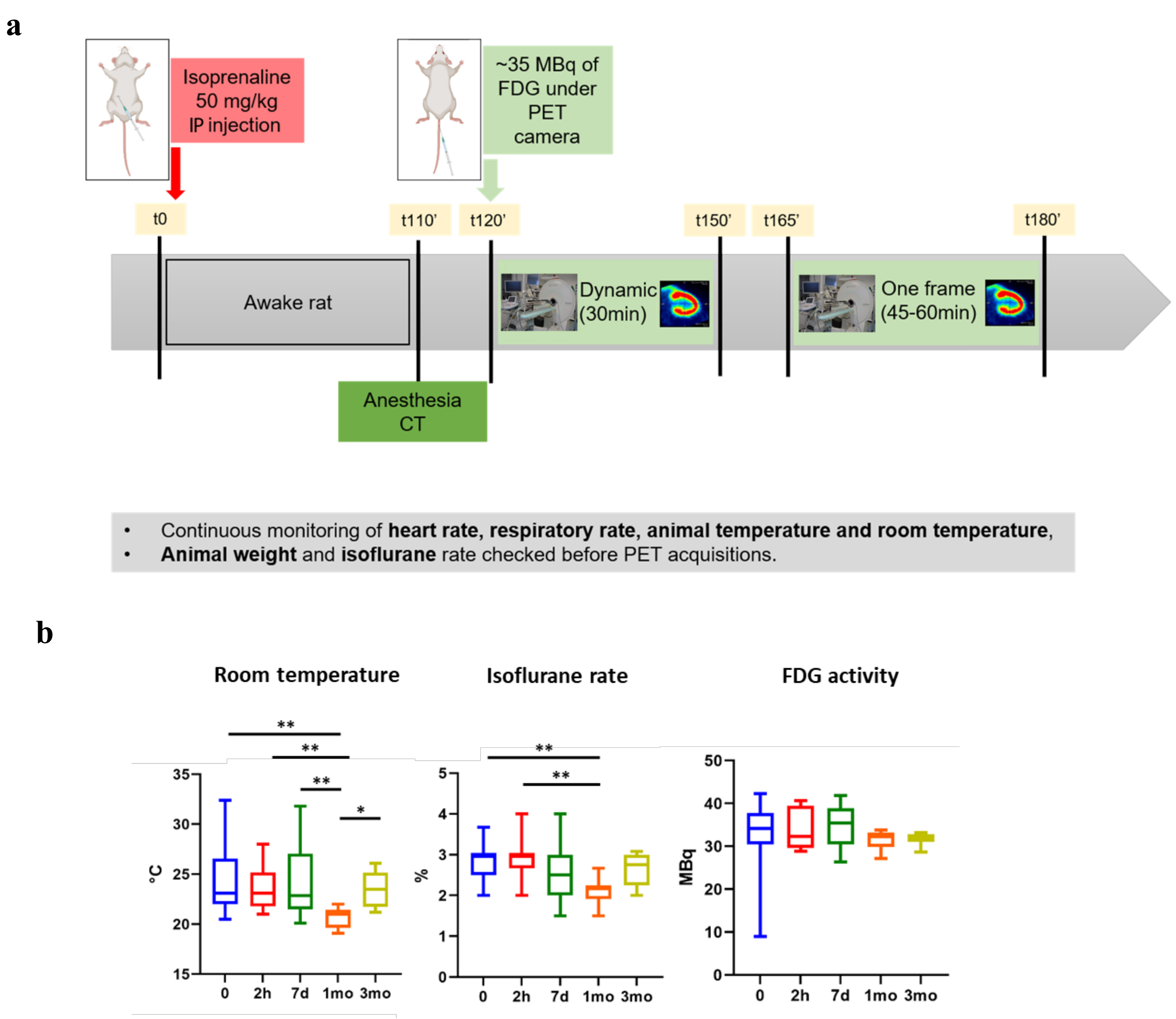

Supplementary figure 7. PET FDG acquisitions. a summarizes the protocol. PET FDG scans were acquired firstly dynamically for 30min after 30s post FDG i.v. injection, followed by 30min static acquisition. **b** Conditions of acquisitions were monitored systematically during the whole scans, such as room temperature (0 vs. 1mo: p = 0.0014, 2h vs. 1mo: p= 0.0070, 7d vs. 1mo: p=0.0072, and 1mo vs. 3mo: p=0.0429) and isoflurane rate (0 vs. 1mo: p = 0.0084 and 2h vs. 1mo: p= 0.0014), here represented in boxplots showing median, 25 and 75 percentiles, and extremes of values. No changes in injected FDG radioactivity were observed between the groups. Unpaired comparison tests: * p<0.05, ** p<0.01 and *** p<0.001. n=18 animals in groups from 0 to 7d post-ISO and n=9 in groups 1mo and 3mo post-ISO.

a

Number of proteins identified by MS proteomics and of proteins used for group comparisons		
(L1)	Total number of identified proteins across all samples	4257
(L2)	Among (A), proteins with at least one LFQ	3203
(L3)	Among (B), proteins with at least 3 out of 5 LFQ in at least one group	2618
(L4)	Proteins with a LFQ in each sample	971

b **1D principal component analysis related to proteins with 100% of valid values**

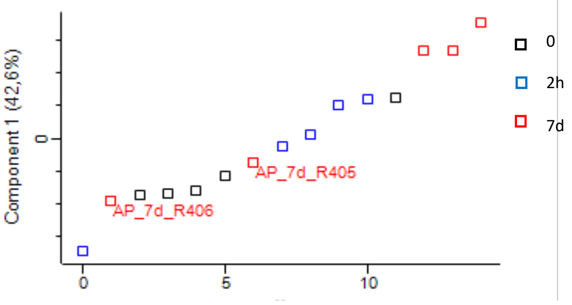

Supplementary figure 8. Proteomic analysis.

a Number of proteins identified by MS proteomics used for group comparisons, indicating the levels (L) of stringency, L1< L2 <L3 < L4. L1. Unfiltered data from the LFQ intensity columns of the *proteingroup.txt* file of MaxQuant output, corresponding to proteins identified with an intensity value in at least one sample, yet without necessarily a sufficient level of confidence and correlation across the proteins' distinct peptide intensities to qualify for quantification. L2: Proteins bearing at least one value in the LFQ intensity columns of the *proteingroup.txt* file of MaxQuant output. L3: Proteins filtered to show enough valid values for statistical analysis (3/5 samples in at least one group). These are the proteins listed in the table of Supplementary table 1. L4: Proteins with an LFQ value in every sample. **b** 1D representation of the component 1 of the principal component analysis (PCA) related to proteins with 100% of valid values (proteins that have LFQ intensity value for all conditions) in the LV apex. The two apical 7d post-ISO "outliers" (R405 and R406 in red) showed a lower performance in protein identifications with around 25% less identifications compared to all other samples and are located in the control side (in black) while the three apical samples (in red) of the five 7d post-ISO samples distinguished from the control and 2h post-ISO groups, indicating a clear post-ISO phenotype.

Canonical Pathways involved in apical metabolism remodeling	2h vs 0			7d vs 0		
	p-value	z-score	involved proteins	p-value	z-score	involved proteins
Insulin Secretion Signaling Pathway	1.04E-01	1.342	EIF4G2,PABPC1,PLCB3,RAP1B,SRP54	3.03E-01	2.828	ADCY6,CAMK2D,CAMK2G,EIF4G2,EIF4G3,MTOR,PABPC1,PLCB3
Insulin Receptor Signaling	4.20E-01	NaN	AKT2,RAP1B	1.60E-01	1.633	AKT2,GYS1,INPPL1,MTOR,PPP1CB,Ppp1cc
PI3K/AKT Signaling	4.37E-02	0.000	AKT2,ELP1,PPP2R2A,RAP1B,THEM4	1.02E-02	1.000	AKT2,GYS1,INPPL1,ITGA6,MTOR,PPP2R3A,PPP2R5E,THEM4,YWHAE,YWHAQ,YWHAZ
14-3-3-mediated Signaling	1.39E-01	NaN	AKT2,PLCB3,RAP1B	1.13E-01	2.236	AKT2,PLCB3,TUBA1C,YWHAE,YWHAQ,YWHAZ
3-phosphoinositide Degradation	1.00E+00	NaN	PDXP	2.23E-01	2.236	ALPL,INPPL1,PALD1,Ppp1cc,PPP2R3A,PPP2R5E
3-phosphoinositide Biosynthesis	1.00E+00	NaN	PDXP	4.39E-01	2.000	ALPL,PALD1,Ppp1cc,PPP2R3A,PPP2R5E
D-myo-inositol-5-phosphate Metabolism	4.78E-01	NaN	PDXP,PLCB3	2.27E-01	2.236	ALPL,PALD1,PLCB3,Ppp1cc,PPP2R3A,PPP2R5E
Superpathway of Inositol Phosphate Compounds	1.00E+00	NaN	PDXP,PLCB3	2.68E-01	2.449	ALPL,INPPL1,PALD1,PLCB3,Ppp1cc,PPP2R3A,PPP2R5E
D-myo-inositol (3,4,5,6)-tetrakisphosphate Biosynthesis	1.00E+00	NaN	PDXP	3.08E-01	2.000	ALPL,PALD1,Ppp1cc,PPP2R3A,PPP2R5E
D-myo-inositol (1,4,5,6)-Tetrakisphosphate Biosynthesis	1.00E+00	NaN	PDXP	3.08E-01	2.000	ALPL,PALD1,Ppp1cc,PPP2R3A,PPP2R5E
AMPK Signaling	1.84E-01	NaN	AKT2,PPP2R2A,RAB3A,STIM1	2.82E-02	0.816	AK2,AKT2,GYS1,MTOR,PFKM,PFKP,PPM1E,PPP2R3A,PPP2R5E,PRKAB1,STIM1
Glycolysis I	_			4.47E-03	-1.000	GAPDH,PFKM,PFKP,PGAM2
Gluconeogenesis I	2.37E-01	NaN	ME3	4.47E-03	-1.000	GAPDH,MDH1,ME3,PGAM2
Oxidative Phosphorylation	3.80E-03	0.447	COX15,NDUFA3,NDUFB4,NDUFB6,NDUFB8	3.55E-07	-1.604	ATP5F1A,ATP5MC1,ATP5PO,ATPAF1,Cox7a2/Cox7a2l2,CYB5A,NDUFA6,NDUFA8,NDUFA9,NDUFB10,NDUFB4,NDUFB7,NDUFB8,NDUFS7
Fatty Acid β-oxidation I	_	_	_	1.10E-02	NaN	ECHDC3,ECHS1,HADHA,HADHB
Acetyl-CoA Biosynthesis III (from Citrate)	_	_	_	_	_	_
Acetyl-CoA Biosynthesis I (Pyruvate Dehydrogenase Complex)	6.31E-02	NaN	DBT	_	_	_

Symbol	Gene Name	2h vs 0		7d vs 0	
		p-value	Fold Change	p-value	Fold Change
**FABP3**	**fatty acid binding protein 3**			7.08E-03	**-1.554**
**FABP4**	**fatty acid binding protein 4**			1.63E-02	**-1.373**

Supplementary table 1. Analysis of canonical pathways involved in apical metabolism. Shown are significant changes in the canonical pathways as defined by Ingenuity Pathway Analysis software, based on mass spectrometry analysis of tissue extracts from n=5 animals for each time point. Comparative p values and z-scores between baseline (no-ISO) and 2h post-ISO animals, and baseline and 7d post-ISO, respectively. Significantly over-represented terms (canonical pathways, functions, upstream regulators) were identified with a right-tailed Fisher's Exact Test that calculates an overlap p-value. The z-score was also calculated to assess the activation (positive z-score) or repression (negative one) of each term. The mass spectrometry proteomics data have been deposited to the ProteomeXchange Consortium via the PRIDE partner repository with the dataset identifier PXD032667.

Canonical Pathways involved in apical tissue remodeling	2h vs. 0			7d vs. 0		
	p-value	z-score	involved proteins	p-value	z-score	involved proteins
Cardiac β-adrenergic Signaling	1.00E+00	NaN	PPP2R2A	3.09E-02	-0.816	ADCY6,AKAP1,ATP2A2,PDE2A,PPP1CB,Ppp1cc,PPP2R3A,PPP2R5E
cAMP-mediated signaling	_	_	_	3.92E-01	2.449	ADCY6,AKAP1,CAMK1,CAMK2D,CAMK2G,GNAI2,PDE2A
mTOR Signaling	1.29E-06	1.000	AKT2,Cdc42,EIF3A,EIF3I,EIF4G2,PPP2R2A,RAP1B,RHOT2,RPS11,RPS12,RPS16,RPS18	2.69E-05	1.134	AKT2,Cdc42,DGKZ,EIF3A,EIF3H,EIF3I,EIF3M,EIF4G2,EIF4G3,MTOR,PPP2R3A,PPP2R5E,PRKAB1,RHOT1,RHOT2,RPS21,RPS8
p70S6K Signaling	1.15E-02	1.342	AKT2,F2,PLCB3,PPP2R2A,RAP1B	7.59E-03	1.342	AKT2,GNAI2,MTOR,PLCB3,PPP2R3A,PPP2R5E,YWHAE,YWHAQ,YWHAZ
Regulation of eIF4 and p70S6K Signaling	8.91E-07	NaN	AKT2,EIF3A,EIF3I,EIF4G2,PABPC1,PPP2R2A,RAP1B,RPS11,RPS12,RPS16,RPS18	9.12E-05	NaN	AKT2,EIF3A,EIF3H,EIF3I,EIF3M,EIF4G2,EIF4G3,ITGA6,MTOR,PABPC1,PPP2R3A,PPP2R5E,RPS21,RPS8
Role of NFAT in Cardiac Hypertrophy	3.85E-01	NaN	AKT2,PLCB3,RAP1B	2.11E-01	2.121	ADCY6,AKT2,CACNA2D2,CAMK1,CAMK2D,CAMK2G,GNAI2,PLCB3
Natural Killer Cell Signaling	1.06E-01	0.000	AKT2,COL18A1,HSPA1A/HSPA1B,RAP1B	3.11E-01	1.633	AKT2,CFL2,COL1A1,HSPA1A/HSPA1B,MTOR,Pak2
IL-6 Signaling	1.07E-02	-1.000	AKT2,CSNK2A1,ELP1,HSPB3,RAP1B	2.50E-01	0.447	A2M,AKT2,COL1A1,CSNK2A1,HSPB3
IL-8 Signaling	1.70E-02	1.000	AKT2,Cdc42,ELP1,RAP1B,RHOT2,ROCK2	7.94E-02	2.121	AKT2,Cdc42,GNAI2,IQGAP1,LASP1,MTOR,MYL2,RHOT1,RHOT2
Phospholipase C Signaling	2.52E-01	NaN	Cdc42,PLCB3,RAP1B,RHOT2	2.06E-01	0.447	ADCY6,Cdc42,ITGA6,MYL2,PEBP1,PLCB3,PPP1CB,RHOT1,RHOT2
HIF1α Signaling	3.41E-01	NaN	AKT2,HSPA1A/HSPA1B,RAP1B	8.32E-02	1.414	AKT2,CAMK1,CAMK2D,CAMK2G,CUL2,HK2,HSPA1A/HSPA1B,MTOR,RAN
Production of Nitric Oxide and Reactive Oxygen Species in Macrophages	2.88E-03	2.000	AKT2,APOA1,Cdc42,ELP1,PPP2R2A,RAP1B,RHOT2	1.10E-01	0.816	AKT2,APOA1,Cdc42,PPP1CB,PPP2R3A,PPP2R5E,RHOT1,RHOT2
HIPPO signaling	2.09E-01	NaN	PPP2R2A,SKP1	3.09E-04	-2.000	DLG1,PPP1CB,Ppp1cc,PPP2R3A,PPP2R5E,SKP1,YWHAE,YWHAQ,YWHAZ
Hepatic Fibrosis Signaling Pathway	1.93E-01	1.633	AKT2,Cdc42,COL18A1,RAP1B,RHOT2,ROCK2	1.00E+00	2.121	AKT2,Cdc42,COL1A1,GNAI2,ITGA6,MTOR,MYL2,RHOT1,RHOT2
Regulation of Actin-based Motility by Rho	2.72E-01	NaN	Cdc42,RHOT2	1.20E-03	1.134	ACTR3,ARPC2,Cdc42,ITGA6,MYL2,Pak2,PPP1CB,RHOT1,RHOT2
Integrin Signaling	1.78E-01	NaN	AKT2,Cdc42,RAP1B,RHOT2	5.13E-05	2.840	ACTN1,ACTN2,ACTN4,ACTR3,AKT2,ARPC2,CAPN1,CAPN2,CAPNS1,Cdc42,CTTN,ITGA6,MYL2,Pak2,PPP1CB,RHOT1,RHOT2
Actin Cytoskeleton Signaling	2.02E-01	1.000	F2,FN1,RAP1B,ROCK2	3.09E-04	1.291	ACTN1,ACTN2,ACTN4,ACTR3,ARPC2,CFL2,FN1,IQGAP1,ITGA6,MYH10,MYH6,MYH9,MYL2,Pak2,PPP1CB,SLC9A1
Actin Nucleation by ARP-WASP Complex	8.32E-03	NaN	Cdc42,RAP1B,RHOT2,ROCK2	1.58E-02	2.000	ACTR3,ARPC2,Cdc42,ITGA6,RHOT1,RHOT2
Glutathione Redox Reactions I	2.20E-01	NaN	GSR	3.31E-03	-2.000	GPX3,GSTM1,GSTM2,MGST3
Glutathione-mediated Detoxification	2.69E-01	NaN	LANCL1	7.76E-03	-1.000	ANPEP,GSTM1,GSTM2,MGST3
Apelin Cardiomyocyte Signaling Pathway	2.72E-01	NaN	AKT2,PLCB3	4.68E-02	0.816	AKT2,ATP2A2,GNAI2,MYL2,PLCB3,SLC9A1
Apelin Endothelial Signaling Pathway	1.20E-01	NaN	AKT2,PLCB3,RAP1B	8.91E-02	1.633	ADCY6,AKT2,GNAI2,MTOR,PLCB3,PRKAB1
Xenobiotic Metabolism General Signaling Pathway	4.42E-01	NaN	AKT2,RAP1B	3.35E-01	-1.342	AKT2,CUL3,GSTM1,GSTM2,MGST3
Xenobiotic Metabolism PXR Signaling Pathway	1.00E+00	NaN	ALDH16A1	8.51E-03	-1.508	ALDH16A1,ALDH2,ALDH6A1,CAMK2D,CAMK2G,GSTM1,GSTM2,MAOA,MGST3,PPP1CB,Ppp1cc
Xenobiotic Metabolism CAR Signaling Pathway	5.55E-01	NaN	ALDH16A1,PPP2R2A	1.02E-01	-2.121	ALDH16A1,ALDH2,ALDH6A1,GSTM1,GSTM2,MGST3,PPP2R3A,PPP2R5E
Xenobiotic Metabolism AHR Signaling Pathway	5.75E-01	NaN	ALDH16A1	2.14E-02	-2.449	ALDH16A1,ALDH2,ALDH6A1,GSTM1,GSTM2,MGST3
Adrenomedullin signaling pathway	5.25E-02	0.447	AKT2,C3,CFH,PLCB3,RAP1B	1.00E+00	2.000	ADCY6,AKT2,CFH,PLCB3
Estrogen Receptor Signaling	4.33E-01	0.000	AKT2,PLCB3,RAP1B,ROCK2	1.53E-01	1.155	ADCY6,AKT2,ATP5F1A,ATP5MC1,CFL2,GNAI2,MTOR,MYL2,PLCB3,PPP1CB,PRKAB1,TFAM
GNRH Signaling	5.38E-01	NaN	PLCB3,RAP1B	1.77E-01	2.449	ADCY6,CACNA2D2,CAMK2D,CAMK2G,GNAI2,Pak2,PLCB3

Supplementary Table 2. Analysis of canonical pathways involved in apical tissue remodeling. Shown are significant changes in the canonical pathways implicated in apical tissue remodeling, as defined by Ingenuity Pathway Analysis software, based on mass spectrometry analysis of tissue extracts from n=5 animals for each time point. Comparative p values and z-scores between baseline (no-ISO) and 2h post-ISO animals, and baseline and 7d post-ISO, respectively. Significantly over-represented terms (canonical pathways, functions, upstream regulators) were identified with a right-tailed Fisher's Exact Test that calculates an overlap p-value. The z-score was also calculated to assess the activation (positive z-score) or repression (negative one) of each term. The mass spectrometry proteomics data have been deposited to the ProteomeXchange Consortium via the PRIDE partner repository with the dataset identifier PXD032667.

Canonical Pathways involved in apical vascular remodeling	2h vs. 0			7d vs. 0		
	p-value	z-score	involved proteins	p-value	z-score	involved proteins
VEGF Signaling	8.51E-02	NaN	AKT2,RAP1B,ROCK2	1.25E-01	1.000	ACTN1,ACTN2,ACTN4,AKT2,YWHAE
P2Y Purigenic Receptor Signaling Pathway	1.47E-01	NaN	AKT2,PLCB3,RAP1B	4.39E-01	1.000	ADCY6,AKT2,GNAI2,PLCB3
P2Y Purigenic Receptor Signaling Pathway	1.47E-01	NaN	AKT2,PLCB3,RAP1B	4.39E-01	1.000	ADCY6,AKT2,GNAI2,PLCB3
Coagulation System	1.51E-06	0.816	F2,FGA,FGG,PLG,SERPIND1,SERPINF2	2.40E-01	NaN	A2M,F13A1

Supplementary Table 3. Analysis of canonical pathways involved in apical vascular remodeling. Shown are significant changes in the canonical pathways implicated apical vascular remodeling, as defined by Ingenuity Pathway Analysis software, based on mass spectrometry analysis of tissue extracts from n=5 animals for each time point. Comparative p values and z-scores between baseline (no-ISO) and 2h post-ISO animals, and baseline and 7d post-ISO, respectively. Significantly over-represented terms (canonical pathways, functions, upstream regulators) were identified with a right-tailed Fisher's Exact Test that calculates an overlap p-value. The z-score was also calculated to assess the activation (positive z-score) or repression (negative one) of each term. The mass spectrometry proteomics data have been deposited to the ProteomeXchange Consortium via the PRIDE partner repository with the dataset identifier PXD032667.

Canonical Pathways involved in basal metabolic remodeling	2h vs. 0			7d vs. 0		
	p-value	z-score	Involved proteins	p-value	z-score	Involved proteins
Insulin Receptor Signaling	4.57E-03	0.816	AKT2,GSK3A,INPPL1,MAP2K2,MAPK3,PPP1R7	1.86E-01	NaN	INPPL1,PPP1R7,SLC2A4,TRIP10,VAMP2
Insulin Secretion Signaling Pathway	1.31E-01	0.447	MAPK3,NSF,SRP54,SRP68,YES1	1.70E-02	1.508	CAMK2G,EIF2A,EIF2S2,EIF4G3,MAPK14,SLC2A4,SRP14,SSR1,STAT5B,VAMP2,YES1
Oxidative Phosphorylation	2.57E-02	1	ATP5PF,COX6A2,DMAC2L,NDUFB6	6.31E-03	0.378	ATP5PF,COX6B1,NDUFA5,NDUFAB1,NDUFB4,NDUFB8,NDUFS7

Supplementary Table 4. Analysis of canonical pathways involved in basal metabolic remodeling. Shown are significant changes in the canonical pathways implicated in basal metabolic remodeling, as defined by Ingenuity Pathway Analysis software, based on mass spectrometry analysis of tissue extracts from n=5 animals for each time point. Comparative p values and z-scores between baseline (no-ISO) and 2h post-ISO animals, and baseline and 7d post-ISO, respectively. Significantly over-represented terms (canonical pathways, functions, upstream regulators) were identified with a right-tailed Fisher's Exact Test that calculates an overlap p-value. The z-score was also calculated to assess the activation (positive z-score) or repression (negative one) of each term. The mass spectrometry proteomics data have been deposited to the ProteomeXchange Consortium via the PRIDE partner repository with the dataset identifier PXD032667.

Canonical Pathways involved in basal vascular remodeling	2h vs. 0			7d vs. 0		
	p-value	z-score	Involved proteins	p-value	z-score	Involved proteins
P2Y Purigenic Receptor Signaling Pathway	1.45E-02	1	AKT2,GNAI3,GNB2,MAP2K2,MAPK3	#N/A	_	#N/A
Coagulation System	7.08E-03	NaN	F9,FGA,FGG	1.81E-01	NaN	A2M,F13A1
Thrombin Signaling	8.32E-02	2	AKT2,GNAI3,GNB2,MAP2K2,MAPK3	1.00E+00	NaN	CAMK2G,MAPK14,Ppp1r12a
Intrinsic Prothrombin Activation Pathway	9.55E-04	2	COL18A1,F9,FGA,FGG	2.14E-01	NaN	COL1A1,F13A1

Supplementary Table 5. Analysis of canonical pathways involved in basal vascular remodeling. Shown are significant changes in the canonical pathways implicated in basal vascular remodeling, as defined by Ingenuity Pathway Analysis software, based on mass spectrometry analysis of tissue extracts from n=5 animals for each time point. Comparative p values and z-scores between baseline (no-ISO) and 2h post-ISO animals, and baseline and 7d post-ISO, respectively. Significantly over-represented terms (canonical pathways, functions, upstream regulators) were identified with a right-tailed Fisher's Exact Test that calculates an overlap p-value. The z-score was also calculated to assess the activation (positive z-score) or repression (negative one) of each term. The mass spectrometry proteomics data have been deposited to the ProteomeXchange Consortium via the PRIDE partner repository with the dataset identifier PXD032667.

Canonical Pathways involved in basal tissue remodeling	2h vs. 0			7d vs. 0		
	p-value	z-score	Involved proteins	p-value	z-score	Involved proteins
**Cardiac β-adrenergic Signaling**	4.58E-01	NaN	GNB2,PPP1R7	3.58E-01	-1	APEX1,ATP2A2,PPP1R7,PPP2R5D
**PI3K/AKT Signaling**	4.79E-03	0.447	AKT2,ELP1,GSK3A,INPPL1,ITGA6,MAP2K2,MAPK3	1.00E+00	NaN	INPPL1,ITGA6,PPP2R5D,YWHAE
**mTOR Signaling**	2.57E-02	NaN	AKT2,MAPK3,RPS16,RPS21,RPS6,RPS9	1.35E-02	NaN	EIF3A,EIF3B,EIF3C,EIF3I,EIF3M,EIF4B,EIF4G3,PPP2R5D,PRKAA2,RPS16
**p70S6K Signaling**	3.39E-03	2.449	AGT,AKT2,GNAI3,MAP2K2,MAPK3,RPS6	1.00E+00	NaN	PPP2R5D,YWHAE
**Role of NFAT in Cardiac Hypertrophy**	1.07E-02	0.447	AKT2,GNAI3,GNB2,HDAC2,MAP2K2,MAPK3,MAPK9	1.00E+00	NaN	CAMK2G,MAPK14
**Natural Killer Cell Signaling**	4.57E-02	1.342	AKT2,COL18A1,HSPA1A/HSPA1B,MAP2K2,MAPK3	1.00E+00	NaN	COL1A1,HSPA1A/HSPA1B,MAPK14
**IL-6 Signaling**	1.45E-02	1	AKT2,ELP1,MAP2K2,MAPK3,MAPK9	3.07E-01	1	A2M,COL1A1,HSPB3,MAPK14
**IL-8 Signaling**	6.76E-03	1.342	AKT2,ELP1,GNAI3,GNB2,MAP2K2,MAPK3,MAPK9	#N/A	_	#N/A
**3-phosphoinositide Degradation**	2.95E-02	0.447	ACP6,INPPL1,NUDT5,PPP1R7,PTPMT1	1.22E-01	0.816	EPHX2,INPPL1,NUDT5,PPP1R7,PPP2R5D,PRUNE1
**D-myo-inositol (1,4,5)-Trisphosphate Biosynthesis**	#N/A	_	#N/A	#N/A	_	#N/A
**Production of Nitric Oxide and Reactive Oxygen Species in Macrophages**	4.37E-03	0.816	AKT2,APOA1,APOM,ELP1,MAPK3,MAPK9,PPP1R7	5.66E-01	0	APOA1,MAPK14,PPP1R7,PPP2R5D
**Regulation of Actin-based Motility by Rho**	1.00E+00	NaN	ITGA6	3.67E-01	NaN	ARPC3,ITGA6,Ppp1r12a
**Integrin Signaling**	2.12E-01	2	AKT2,ITGA6,MAP2K2,MAPK3	4.95E-01	1	ACTN2,ARF3,ARPC3,ITGA6,Ppp1r12a
**Glutathione Redox Reactions I**	#N/A	_	#N/A	1.48E-02	NaN	GPX3,GSTM1,GSTM2
**Apelin Cardiomyocyte Signaling Pathway**	2.45E-02	1	AKT2,GNAI3,MAPK3,MAPK9	1.00E+00	NaN	ATP2A2,MAPK14
**Apelin Endothelial Signaling Pathway**	9.77E-03	1.342	AKT2,GNAI3,MAP2K2,MAPK3,MAPK9	1.00E+00	NaN	PRKAA2
**Xenobiotic Metabolism General Signaling Pathway**	5.62E-03	0.816	AKT2,CUL3,GCLC,MAP2K2,MAPK3,MAPK9	1.00E+00	NaN	GSTM1,GSTM2,MAPK14
**Xenobiotic Metabolism PXR Signaling Pathway**	1.42E-01	-1	ALDH16A1,ALDH1A1,ALDH3A2,PPP1R7	1.00E-01	-1.134	ALDH1A1,CAMK2G,GSTM1,GSTM2,MAOB,PPM1A,PPP1R7
**Xenobiotic Metabolism CAR Signaling Pathway**	5.01E-02	-2.236	ALDH16A1,ALDH1A1,ALDH3A2,MAP2K2,MAPK3	5.52E-01	-1	ALDH1A1,GSTM1,GSTM2,PPP2R5D
**Adrenomedullin signaling pathway**	6.46E-03	0.378	AKT2,C3,CFH,GSK3A,MAP2K2,MAPK3,MAPK9	1.00E+00	NaN	MAPK14

Supplementary Table 6. Analysis of canonical pathways involved in basal tissue remodeling. Shown are significant changes in the canonical pathways implicated in basal tissue remodeling, as defined by Ingenuity Pathway Analysis software, based on mass spectrometry analysis of tissue extracts from n=5 animals for each time point. Comparative p values and z-scores between baseline (no-ISO) and 2h post-ISO animals, and baseline and 7d post-ISO, respectively. Significantly over-represented terms (canonical pathways, functions, upstream regulators) were identified with a right-tailed Fisher's Exact Test that calculates an overlap p-value. The z-score was also calculated to assess the activation (positive z-score) or repression (negative one) of each term. The mass spectrometry proteomics data have been deposited to the ProteomeXchange Consortium via the PRIDE partner repository with the dataset identifier PXD032667.
