# Supplementary material for: Acute stress induces long-term metabolic, functional, and structural remodeling of the heart
Source: Nat Commun. 2023 Jun 28;14:3835. doi: 10.1038/s41467-023-39590-3 (PMC10307856; doi:10.1038/s41467-023-39590-3)
Supplement: Supplementary file 3 — Reporting Summary [file 41467_2023_39590_MOESM3_ESM.pdf]

nature portfolio

Corresponding author(s): Bertrand Tavitian & Thulaciga Yoganathan

Last updated by author(s): May 25, 2023

Reporting Summary

Nature Portfolio wishes to improve the reproducibility of the work that we publish. This form provides structure for consistency and transparency in reporting. For further information on Nature Portfolio policies, see our Editorial Policies and the Editorial Policy Checklist.

Statistics

For all statistical analyses, confirm that the following items are present in the figure legend, table legend, main text, or Methods section.

n/a	Confirmed	
☐	☒	The exact sample size (*n*) for each experimental group/condition, given as a discrete number and unit of measurement
☐	☒	A statement on whether measurements were taken from distinct samples or whether the same sample was measured repeatedly
☐	☒	The statistical test(s) used AND whether they are one- or two-sided<br>*Only common tests should be described solely by name; describe more complex techniques in the Methods section.*
☐	☒	A description of all covariates tested
☐	☒	A description of any assumptions or corrections, such as tests of normality and adjustment for multiple comparisons
☐	☒	A full description of the statistical parameters including central tendency (e.g. means) or other basic estimates (e.g. regression coefficient) AND variation (e.g. standard deviation) or associated estimates of uncertainty (e.g. confidence intervals)
☐	☒	For null hypothesis testing, the test statistic (e.g. *F*, *t*, *r*) with confidence intervals, effect sizes, degrees of freedom and *P* value noted<br>*Give P values as exact values whenever suitable.*
☒	☐	For Bayesian analysis, information on the choice of priors and Markov chain Monte Carlo settings
☒	☐	For hierarchical and complex designs, identification of the appropriate level for tests and full reporting of outcomes
☐	☒	Estimates of effect sizes (e.g. Cohen's *d*, Pearson's *r*), indicating how they were calculated

Our web collection on statistics for biologists contains articles on many of the points above.

Software and code

Policy information about availability of computer code

Data collection

- Positron Emission Tomography - X-ray tomodensitometry: NanoPETCT, Mediso Medical Imaging Systems, Budapest, Hungary.
- Ultrafast ultrasound imaging: Aixplorer connected to the pediatric ultrasound probe: SuperLinear™ SLH20-6 (central frequency 15 MHz), both from Supersonic Imagine, Aix-en-Provence, France.
- High resolution 2D echocardiography: 18 MHz, VEVO2100, Visualsonics, Toronto, Canada.
- Cardiac Magnetic Resonance imaging: preclinical 4.7 T MRI system, Bruker BioSpec47/40 USR, Ettlingen, Germany; data were acquired using Paravision software 6.0.1, Bruker, Ettlingen, Germany.
- Electrocardiogram: PowerLab, AdInstruments, Sydney, Australia.
- Noninvasive blood pressure measurement: tail-cuff CODA® High Throughput system, Kent Scientific Corporation, Torrington, CT, USA.
- Digital scan of colored sections: NanoZoomer HT 2.0 (Hamamatsu, Hamamatsu City, Japan) at a magnification of x20.
- Digital scan of immune-stained sections: Vectra® Polaris™ (AKOYA Biosciences, Menlo Park, CA, USA).
- Second Harmonic Generation images: onlinear multiphoton Leica SP8 DIVE microscope (Leica microsystems Gmbh, Wetzlar, Germany). coupled with a Coherent Discovery (Coherent Inc., Santa Clara, CA, USA) laser source.
- Ultrastructure imaging of the heart: transmission electron microscope JEOL, JEOL ltd, Tokyo, Japan. Images acquired with the camera GATAN Orius and processed using Digital Micrograph® software, both from AMETEK, USA.
- Proteomic data collection: ProtiFi, NY, USA. Otof control software from Bruker Daltonik GmbH, Bremen, Germany.

Data analysis

The two softwares developed for this study have been made freely available:
- Fiber-ML is available at https://gitlab.com/balvayda/fiber-ml
- CardioTrack is available at https://gitlab.com/LIB_ICV/cardiotrack

- Reconstruction of FDG PET data: 3D-OSEM using Teratomo package from Mediso, Budapest, Hungary.
- Analysis of FDG PET data: PMOD software (version 3.8) from PMOD Technologies Ltd, Zürich, Switzerland
- Compartmental kinetic assessment of FDG uptake: PMOD kinetics package (version 3.8, PMOD Technologies Ltd, Zürich, Switzerland).
- Cardiac PETRUS analysis: Perez-Liva, M. et al. Ultrafast Ultrasound Imaging for Super-resolution Preclinical Cardiac PET. Mol. Imaging Biol. (2020) doi:DOI: 10.1007/s11307-020-01512-w.
- Analysis of MRI T1 cine cardiac images: Circle civ42 software, version 5.13.5, Circle Cardiovascular Imaging Inc., Calgary, Canada.
- Myocardial strain: CardioTrack software, Sorbonne University, Paris, France (Lamy, J. et al. Scan-rescan reproducibility of ventricular and atrial MRI feature tracking strain. Comput. Biol. Med. 92, 197–203 (2018).; Huber, A. T. et al. Cardiac MR Strain: A Noninvasive Biomarker of Fibrofatty Remodeling of the Left Atrial Myocardium. Radiology 286, 83–92, 2018), based on feature tracking (Voigt, J.-U. & Cvijic, M. 2- and 3-Dimensional Myocardial Strain in Cardiac Health and Disease. JACC Cardiovasc. Imaging 12, 1849–1863, 2019), and written in Matlab (The MathWorks, Natick, MA, USA).
- Histological staining quantification and analysis: Fiber-ML, written in Matlab® (The MathWorks, Natick, MA, USA), Facchin, C. et al. FIBER-ML, an Open-Source Supervised Machine Learning Tool for Quantification of Fibrosis in Tissue Sections. Am. J. Pathol. S0002944022000517 (2022) doi:10.1016/j.ajpath.2022.01.013.
- Calculations of the left ventricle's capillary density: ImageJ® .
- Second Harmonic Generation score: ImageJ® .
- Quantification of immunoblots: ImageJ®.
- LC-MS analyses: U3000 RSLC nano-LC (Dionex) system coupled to a TIMS-TOF Pro mass spectrometer (Bruker Daltonik GmbH, Bremen, Germany).
- Analysis of mass spectrometry data: MaxQuant version 1.6.17 (Max Planck institute of Biochemistry, Germany); For differential analysis, LFQ results from MaxQuant were imported into the Perseus software version 1.6.14 (Max-Planck Institute of Biochemistry); over-representation analysis using Ingenuity Pathway Analysis version 60467501 (QIAGEN Inc, Venlo, The Netherlands).
- Statistical analysis: GraphPad Prism 9.2.0 (GraphPad Software, San Diego, CA, USA).

For manuscripts utilizing custom algorithms or software that are central to the research but not yet described in published literature, software must be made available to editors and reviewers. We strongly encourage code deposition in a community repository (e.g. GitHub). See the Nature Portfolio guidelines for submitting code & software for further information.

Data

Policy information about availability of data

All manuscripts must include a data availability statement. This statement should provide the following information, where applicable:

- Accession codes, unique identifiers, or web links for publicly available datasets
- A description of any restrictions on data availability
- For clinical datasets or third party data, please ensure that the statement adheres to our policy

The mass spectrometry proteomics data that support the findings of this study are available in the ProteomeXchange Consortium via the PRIDE80 partner repository with the dataset identifier PXD032667.
The authors declare that the rest of the data supporting the findings of this study are available within the paper and its supplementary information files.

Human research participants

Policy information about studies involving human research participants and Sex and Gender in Research.

Reporting on sex and gender	N/A
Population characteristics	N/A
Recruitment	N/A
Ethics oversight	N/A

Note that full information on the approval of the study protocol must also be provided in the manuscript.

Field-specific reporting

Please select the one below that is the best fit for your research. If you are not sure, read the appropriate sections before making your selection.

[x] Life sciences [ ] Behavioural & social sciences [ ] Ecological, evolutionary & environmental sciences

For a reference copy of the document with all sections, see nature.com/documents/nr-reporting-summary-flat.pdf

Life sciences study design

All studies must disclose on these points even when the disclosure is negative.

Sample size	The sample size was determined according to the registry of the laboratory compiling the variation coefficients of each technique.<br>For cardiac PETRUS and FDG PET, a pilot experiment was performed to anticipate the number of animals.

	For transmission electron microscopy and proteomic analysis, the budget available for the study was also taken into account.<br><br>Non-invasive and long-term monitoring of the animals reduces the sample size since each animal is its own control. Physiological parameters (weight, rectal temperature, blood glucose, plasma energy substrate assays, heart and respiratory rates, ECG, biomarkers of heart failure) of the animals were monitored before and at different times after induction of the TTC model in order to detect any possible signs of pain. In some rare cases of non-recovery, humanly euthanasia was performed.
Data exclusions	None
Replication	In order to check the reproducibility of the TTC animal model, ECG and blood glucose were systematically measured in each animal before proceeding with further experiments.<br>Red Sirius, oil-red and PAS histological colorations were done in triplicate.<br>Plasmatic dosages were duplicated.<br>Regarding the myocardial strain measurements, a pilot study was performed to verify the reproducibility of the CardioTrack software.<br>For the non-invasive, in vivo and long-term monitoring of the animals (FDG PET, US, cardiac PETRUS, Coronary flow reserve measurement, cardiac MRI, blood pressure measurement and ECG), values for each animal were normalized to pre-ISO injection baseline values.<br>We confirm that all attempts at replication were successful.
Randomization	Animals were randomly assigned to an experimental group.
Blinding	For image analysis (FDG TEP, cardiac PETRUS, US imaging, cardiac MRI, myocardial strain, histological quantification, SHG), proteomic analysis, immuno-staining, Red Sirius, oil-red and PAS colorations, the investigators were blinded with respect to the status of the animals.

Behavioural & social sciences study design

All studies must disclose on these points even when the disclosure is negative.

Study description	*Briefly describe the study type including whether data are quantitative, qualitative, or mixed-methods (e.g. qualitative cross-sectional, quantitative experimental, mixed-methods case study).*
Research sample	*State the research sample (e.g. Harvard university undergraduates, villagers in rural India) and provide relevant demographic information (e.g. age, sex) and indicate whether the sample is representative. Provide a rationale for the study sample chosen. For studies involving existing datasets, please describe the dataset and source.*
Sampling strategy	*Describe the sampling procedure (e.g. random, snowball, stratified, convenience). Describe the statistical methods that were used to predetermine sample size OR if no sample-size calculation was performed, describe how sample sizes were chosen and provide a rationale for why these sample sizes are sufficient. For qualitative data, please indicate whether data saturation was considered, and what criteria were used to decide that no further sampling was needed.*
Data collection	*Provide details about the data collection procedure, including the instruments or devices used to record the data (e.g. pen and paper, computer, eye tracker, video or audio equipment) whether anyone was present besides the participant(s) and the researcher, and whether the researcher was blind to experimental condition and/or the study hypothesis during data collection.*
Timing	*Indicate the start and stop dates of data collection. If there is a gap between collection periods, state the dates for each sample cohort.*
Data exclusions	*If no data were excluded from the analyses, state so OR if data were excluded, provide the exact number of exclusions and the rationale behind them, indicating whether exclusion criteria were pre-established.*
Non-participation	*State how many participants dropped out/declined participation and the reason(s) given OR provide response rate OR state that no participants dropped out/declined participation.*
Randomization	*If participants were not allocated into experimental groups, state so OR describe how participants were allocated to groups, and if allocation was not random, describe how covariates were controlled.*

Ecological, evolutionary & environmental sciences study design

All studies must disclose on these points even when the disclosure is negative.

Study description	*Briefly describe the study. For quantitative data include treatment factors and interactions, design structure (e.g. factorial, nested, hierarchical), nature and number of experimental units and replicates.*
Research sample	*Describe the research sample (e.g. a group of tagged Passer domesticus, all Stenocereus thurberi within Organ Pipe Cactus National Monument), and provide a rationale for the sample choice. When relevant, describe the organism taxa, source, sex, age range and any manipulations. State what population the sample is meant to represent when applicable. For studies involving existing datasets, describe the data and its source.*

Sampling strategy	*Note the sampling procedure. Describe the statistical methods that were used to predetermine sample size OR if no sample-size calculation was performed, describe how sample sizes were chosen and provide a rationale for why these sample sizes are sufficient.*
Data collection	*Describe the data collection procedure, including who recorded the data and how.*
Timing and spatial scale	*Indicate the start and stop dates of data collection, noting the frequency and periodicity of sampling and providing a rationale for these choices. If there is a gap between collection periods, state the dates for each sample cohort. Specify the spatial scale from which the data are taken*
Data exclusions	*If no data were excluded from the analyses, state so OR if data were excluded, describe the exclusions and the rationale behind them, indicating whether exclusion criteria were pre-established.*
Reproducibility	*Describe the measures taken to verify the reproducibility of experimental findings. For each experiment, note whether any attempts to repeat the experiment failed OR state that all attempts to repeat the experiment were successful.*
Randomization	*Describe how samples/organisms/participants were allocated into groups. If allocation was not random, describe how covariates were controlled. If this is not relevant to your study, explain why.*
Blinding	*Describe the extent of blinding used during data acquisition and analysis. If blinding was not possible, describe why OR explain why blinding was not relevant to your study.*

Did the study involve field work? ☐ Yes ☐ No

Field work, collection and transport

Field conditions	*Describe the study conditions for field work, providing relevant parameters (e.g. temperature, rainfall).*
Location	*State the location of the sampling or experiment, providing relevant parameters (e.g. latitude and longitude, elevation, water depth).*
Access & import/export	*Describe the efforts you have made to access habitats and to collect and import/export your samples in a responsible manner and in compliance with local, national and international laws, noting any permits that were obtained (give the name of the issuing authority, the date of issue, and any identifying information).*
Disturbance	*Describe any disturbance caused by the study and how it was minimized.*

Reporting for specific materials, systems and methods

We require information from authors about some types of materials, experimental systems and methods used in many studies. Here, indicate whether each material, system or method listed is relevant to your study. If you are not sure if a list item applies to your research, read the appropriate section before selecting a response.

Materials & experimental systems

n/a	Involved in the study	
☐	☒	Antibodies
☒	☐	Eukaryotic cell lines
☒	☐	Palaeontology and archaeology
☐	☒	Animals and other organisms
☒	☐	Clinical data
☒	☐	Dual use research of concern

Methods

n/a	Involved in the study	
☒	☐	ChIP-seq
☒	☐	Flow cytometry
☒	☐	MRI-based neuroimaging

Antibodies

Antibodies used	Immunofluorescence: Glut1 (1:200, rabbit monoclonal, clone EPR3915, #ab115730, Abcam); Glut4 (1:500, rabbit polyclonal, #ab33780, Abcam); Cd68 (1:400, mouse monoclonal, clone ED1, #MCA341R, Bio-rad); Cd31 (1:200, goat polyclonal, #AF3628, R&D systems); cyanine 3 coupled-alpha-Sma (mouse monoclonal, clone 1A4, #C6198, Sigma-Aldrich).<br><br>Western blotting, primary antibodies: anti-Gfat1 (1:1000, #D12F4, Cell Signaling Technology); anti-Gapdh (1:1000, #ab8245, Abcam); anti-Gfat2 (1:1000, #ab190966, Abcam); anti-O-linked N-acetylglucosamine (1:2000, #RL2, Invitrogen). Secondary antibodies: HRP-linked anti-rabbit IgG (1:5,000, goat, #4030-05, SouthernBiotech), anti-mouse IgG (1:5000, light chain binding protein, #sc-516102, Santa Cruz Biotechnology), HRP-linked Donkey anti-rabbit IgG (1:5,000, Jackson ImmunoResearch), HRP-linked Donkey anti-mouse IgG (1:5,000, Jackson ImmunoResearch).
Validation	For IF, validation of Glut1, Glut4 and Cd68 antibodies was done according to their cellular distribution. The Opal™ Multiplex immunohistochemistry (IHC) kits (AKOYA Biosciences, Marlborough, MA and Menlo Park, CA, USA) was used to allow simultaneous IHC detection of the three antigens. Gluts 1 and 4 and cd31 antibodies were validated using IF and WB analysis in sunitib-induced cardiotoxic heart tissue (Sourdon J, Facchin C, Certain A, Viel T, Robin B, Lager F, Marchiol C, Balvay D, Yoganathan T, Favier J, Tharaux PL. Sunitinib-induced cardiac hypertrophy and the endothelin axis. Theranostics. 2021;11(8):3830.; Sourdon J, Lager F, Viel T, Balvay D, Moorhouse R, Bennana E, Renault G, Tharaux PL, Dhaun N, Tavitian B. Cardiac metabolic deregulation induced by the tyrosine kinase receptor inhibitor sunitinib is rescued by endothelin receptor antagonism. Theranostics. 2017;7(11):2757.).<br><br>For WB, the intensity of Gfat1, Gfat2 and O-linked N-acetulglucosamine bands was normalized to that of housekeeping protein Gapdh. Alongside our samples, we have added an internal control which is a mixture of brain (5 ug) and pancreas (10 ug) tissue in a total volume of 20 ul as a positive control.

Eukaryotic cell lines

Policy information about cell lines and Sex and Gender in Research

Cell line source(s)	*State the source of each cell line used and the sex of all primary cell lines and cells derived from human participants or vertebrate models.*
Authentication	*Describe the authentication procedures for each cell line used OR declare that none of the cell lines used were authenticated.*
Mycoplasma contamination	*Confirm that all cell lines tested negative for mycoplasma contamination OR describe the results of the testing for mycoplasma contamination OR declare that the cell lines were not tested for mycoplasma contamination.*
Commonly misidentified lines (See ICLAC register)	*Name any commonly misidentified cell lines used in the study and provide a rationale for their use.*

Palaeontology and Archaeology

Specimen provenance	*Provide provenance information for specimens and describe permits that were obtained for the work (including the name of the issuing authority, the date of issue, and any identifying information). Permits should encompass collection and, where applicable, export.*
Specimen deposition	*Indicate where the specimens have been deposited to permit free access by other researchers.*
Dating methods	*If new dates are provided, describe how they were obtained (e.g. collection, storage, sample pretreatment and measurement), where they were obtained (i.e. lab name), the calibration program and the protocol for quality assurance OR state that no new dates are provided.*

☐ Tick this box to confirm that the raw and calibrated dates are available in the paper or in Supplementary Information.

Ethics oversight	*Identify the organization(s) that approved or provided guidance on the study protocol, OR state that no ethical approval or guidance was required and explain why not.*

Note that full information on the approval of the study protocol must also be provided in the manuscript.

Animals and other research organisms

Policy information about studies involving animals; ARRIVE guidelines recommended for reporting animal research, and Sex and Gender in Research

Laboratory animals	Adult Female Wistar rats of 12-week-old Janvier (Le Genest-St-Isle, France) housed in our facility on a 12:12 light-dark cycle, controlled temperature, 25°C, 60% air humidity, and free access to food and water.
Wild animals	None

Reporting on sex	Female animals were used to match the human disease where more than 80% patients are female
Field-collected samples	None
Ethics oversight	French Animal Ethics Committee agreement number 19-064. All protocols were performed by trained certified personnel and followed the ARRIVE Guidelines.

Note that full information on the approval of the study protocol must also be provided in the manuscript.

Clinical data

Policy information about clinical studies

All manuscripts should comply with the ICMJEguidelines for publication of clinical research and a completedCONSORT checklist must be included with all submissions.

Clinical trial registration	*Provide the trial registration number from ClinicalTrials.gov or an equivalent agency.*
Study protocol	*Note where the full trial protocol can be accessed OR if not available, explain why.*
Data collection	*Describe the settings and locales of data collection, noting the time periods of recruitment and data collection.*
Outcomes	*Describe how you pre-defined primary and secondary outcome measures and how you assessed these measures.*

Dual use research of concern

Policy information about dual use research of concern

Hazards

Could the accidental, deliberate or reckless misuse of agents or technologies generated in the work, or the application of information presented in the manuscript, pose a threat to:

No	Yes	
☒	☐	Public health
☒	☐	National security
☒	☐	Crops and/or livestock
☒	☐	Ecosystems
☒	☐	Any other significant area

Experiments of concern

Does the work involve any of these experiments of concern:

No	Yes	
☒	☐	Demonstrate how to render a vaccine ineffective
☒	☐	Confer resistance to therapeutically useful antibiotics or antiviral agents
☒	☐	Enhance the virulence of a pathogen or render a nonpathogen virulent
☒	☐	Increase transmissibility of a pathogen
☒	☐	Alter the host range of a pathogen
☒	☐	Enable evasion of diagnostic/detection modalities
☒	☐	Enable the weaponization of a biological agent or toxin
☒	☐	Any other potentially harmful combination of experiments and agents

ChIP-seq

Data deposition

☐ Confirm that both raw and final processed data have been deposited in a public database such as GEO.

☐ Confirm that you have deposited or provided access to graph files (e.g. BED files) for the called peaks.

Data access links<br>*May remain private before publication.*	*For "Initial submission" or "Revised version" documents, provide reviewer access links. For your "Final submission" document, provide a link to the deposited data.*
Files in database submission	*Provide a list of all files available in the database submission.*

Genome browser session
(e.g. UCSC)

Provide a link to an anonymized genome browser session for "Initial submission" and "Revised version" documents only, to enable peer review. Write "no longer applicable" for "Final submission" documents.

Methodology

Replicates	*Describe the experimental replicates, specifying number, type and replicate agreement.*
Sequencing depth	*Describe the sequencing depth for each experiment, providing the total number of reads, uniquely mapped reads, length of reads and whether they were paired- or single-end.*
Antibodies	*Describe the antibodies used for the ChIP-seq experiments; as applicable, provide supplier name, catalog number, clone name, and lot number.*
Peak calling parameters	*Specify the command line program and parameters used for read mapping and peak calling, including the ChIP, control and index files used.*
Data quality	*Describe the methods used to ensure data quality in full detail, including how many peaks are at FDR 5% and above 5-fold enrichment.*
Software	*Describe the software used to collect and analyze the ChIP-seq data. For custom code that has been deposited into a community repository, provide accession details.*

Flow Cytometry

Plots

Confirm that:

☐ The axis labels state the marker and fluorochrome used (e.g. CD4-FITC).

☐ The axis scales are clearly visible. Include numbers along axes only for bottom left plot of group (a 'group' is an analysis of identical markers).

☐ All plots are contour plots with outliers or pseudocolor plots.

☐ A numerical value for number of cells or percentage (with statistics) is provided.

Methodology

Sample preparation	*Describe the sample preparation, detailing the biological source of the cells and any tissue processing steps used.*
Instrument	*Identify the instrument used for data collection, specifying make and model number.*
Software	*Describe the software used to collect and analyze the flow cytometry data. For custom code that has been deposited into a community repository, provide accession details.*
Cell population abundance	*Describe the abundance of the relevant cell populations within post-sort fractions, providing details on the purity of the samples and how it was determined.*
Gating strategy	*Describe the gating strategy used for all relevant experiments, specifying the preliminary FSC/SSC gates of the starting cell population, indicating where boundaries between "positive" and "negative" staining cell populations are defined.*

☐ Tick this box to confirm that a figure exemplifying the gating strategy is provided in the Supplementary Information.

Magnetic resonance imaging

Experimental design

Design type	Assessment of heart functions and strain measurements
Design specifications	We followed longitudinally the same exactly 6 rats at 0 (baseline), 2h, and 7d post-ISO. Each animal was its own control throughout the successive time points, which allows a better statistical robustness of the variability of quantitative parameters
Behavioral performance measures	*State number and/or type of variables recorded (e.g. correct button press, response time) and what statistics were used to establish that the subjects were performing the task as expected (e.g. mean, range, and/or standard deviation across subjects).*

Acquisition

Imaging type(s)	Functional
Field strength	4.7 T
Sequence & imaging parameters	LV function was measured by cine T1-weighted cine sequences including short axis stack of 6 to 7 slices covering the left and right ventricles from base to apex, along with conventional 2-chamber and a 4-chamber views, using the following scan parameters: TR=7.2 ms; TE=2.7 ms; flip angle=18.0°; field of view (FOV) = 60 x 60 mm; matrix size = 256 x 256, planar resolution = 235 µm, oversampling = 100, number of frames = 16 per heart cycle, slice thickness= 2 mm. Total scan time was 1min32s per slice.
Area of acquisition	Heart

Diffusion MRI ☐ Used ☒ Not used

Preprocessing

Preprocessing software	*Provide detail on software version and revision number and on specific parameters (model/functions, brain extraction, segmentation, smoothing kernel size, etc.).*
Normalization	*If data were normalized/standardized, describe the approach(es): specify linear or non-linear and define image types used for transformation OR indicate that data were not normalized and explain rationale for lack of normalization.*
Normalization template	*Describe the template used for normalization/transformation, specifying subject space or group standardized space (e.g. original Talairach, MNI305, ICBM152) OR indicate that the data were not normalized.*
Noise and artifact removal	*Describe your procedure(s) for artifact and structured noise removal, specifying motion parameters, tissue signals and physiological signals (heart rate, respiration).*
Volume censoring	*Define your software and/or method and criteria for volume censoring, and state the extent of such censoring.*

Statistical modeling & inference

Model type and settings	*Specify type (mass univariate, multivariate, RSA, predictive, etc.) and describe essential details of the model at the first and second levels (e.g. fixed, random or mixed effects; drift or auto-correlation).*
Effect(s) tested	*Define precise effect in terms of the task or stimulus conditions instead of psychological concepts and indicate whether ANOVA or factorial designs were used.*

Specify type of analysis: ☐ Whole brain ☐ ROI-based ☐ Both

Statistic type for inference (See Eklund et al. 2016)	*Specify voxel-wise or cluster-wise and report all relevant parameters for cluster-wise methods.*
Correction	*Describe the type of correction and how it is obtained for multiple comparisons (e.g. FWE, FDR, permutation or Monte Carlo).*

Models & analysis

n/a	Involved in the study
☒	☐ Functional and/or effective connectivity
☒	☐ Graph analysis
☒	☐ Multivariate modeling or predictive analysis
